# Induction of M2 Polarization in Primary Culture Liver Macrophages from Rats with Acute Pancreatitis

**DOI:** 10.1371/journal.pone.0108014

**Published:** 2014-09-26

**Authors:** Lixia Xu, Fen Yang, Rong Lin, Chaoqun Han, Jun Liu, Zhen Ding

**Affiliations:** Department of Gastroenterology, Union Hospital, Tongji Medical College, Huazhong University of Science and Technology, Wuhan, China; University of Valencia, Spain

## Abstract

**Background and Aims:**

Systemic inflammatory response syndrome (SIRS), a major process of severe acute pancreatitis (SAP), usually occurs after various activated proinflammatory cytokines, which are produced by macrophages such as liver macrophages. Macrophages can secrete not only proinflammatory mediators but also inhibitory inflammatory cytokines such as IL-10, leading to two different functional states defined as “polarization”. The main purpose of this study was to demonstrate the polarization of liver macrophages during severe acute pancreatitis and to explore whether the polarization of these activated Liver macrophages could be reversed in vitro.

**Methods:**

Liver macrophages were isolated from rats with acute pancreatitis. These primary culture macrophages were treated with IL-4 or regulatory T cells in vitro to reverse their polarization and was evaluated by measuring M1/M2 marker expression using real time PCR and immunofluorescence staining.

**Results:**

Acute pancreatitis was induced successfully by intra-pancreatic ductal injection of 5% sodium taurocholate. The liver macrophages demonstrated M1 polarization from 4 h to 16 h after the onset of acute pancreatitis. However, after IL-4 or Treg treatment, the polarization of the liver macrophages was reversed as indicated by increased expression of M2 markers and reduced expression of M1 markers. Furthermore, the effect of Treg on modulating macrophage polarization was slightly better than that of IL-4 in vitro.

**Conclusion:**

Liver macrophages, a pivotal cell type in the pathogenesis of SAP, become M1 polarized during pancreatic inflammation. Treatment of these cells with IL-4 and Treg can reverse this activation in vitro. This method of altering macrophage polarization could be a prospective therapy for SAP.

## Background

Severe acute pancreatitis (SAP) is a life threatening disease especially when multi-remote organs and systems are involved in the inflammation. Systemic inflammatory response syndrome (SIRS) is an essential process during the occurrence of multi-organ failure which contributes to nearly half of deaths in SAP patients [1]; [2].

The over-activation of proinflammatory mediators is generally accepted to play an indispensable role in the development of SIRS. Several vital cytokines and chemokines, such as interleukin (IL)-1, IL-6, tumor necrosis factor (TNF)-α, monocyte chemoattractant protein (MCP)-1, may contribute to pancreatic necrosis and inflammatory cell proliferation [3]; [4]; [5]. Recently, several reports have demonstrated that macrophages can be stimulated during the development of acute pancreatitis and these activated cells become the main source of proinflammatory mediators [6]; [7]. In particular, liver macrophages can be strongly activated in the blood stream with numerous inflammatory mediators including pancreatic necrotizing tissue and lipopolysaccharides (LPS) [8]; [9]. Activated liver macrophages are hypothesized to be heavily involved the early pancreatic inflammation because of their ability to release much more proinflammatory cytokines and mediators to amplify systemic inflammation.

Macrophages can be activated by different pathways and these alternative activated statuses are defined as “M1/M2 polarization” [10]; [11]; [12]. The initial M1 polarization state of macrophages can be induced by interferon (IFN)-γ or LPS, and these cells can also produce and release proinflammatory mediators such as TNF-α, IL-1β and IL-6. The involvement of liver macrophages in acute pancreatitis is a typical response following their M1 polarization. In contrast, M2 polarization of macrophages, initiated by IL-4 or IL-13, results in the synthesis of anti-inflammatory cytokines such as TGF-β_1_ and IL-10 [13]; [14]. M2 macrophages often contribute to the resolution of inflammation and extracellular matrix synthesis during tissue repair [15]; [16].

Liver macrophages usually contribute to the occurrence and amplification of systemic inflammation after the development of acute pancreatitis [17]; [18]. However, there are few studies that confirm which polarization of liver macrophages occurs in the process of acute pancreatitis and its relationship with the severity of this disease.

In this work, we used the SAP model induced by the intraductal injection of sodium taurocholate, and we isolated liver macrophages to investigate their polarization. Furthermore, we treated the primary cultured liver macrophages with IL-4, a well-known inductor of M2 phenotype, to demonstrate that the M1 phenotype of these macrophages can be reversed to the M2 phenotype which favors inflammation resolution. Because of its short half-life IL-4 is difficult to be used in vivo in inhibition of inflammation. We hypothesized that Treg may take better effect on inflammatory suppression for it can secret IL-4 continuously. In our study, we try to use Treg to reverse M1 phenotype of macrophages and compare its effect with that of IL-4.

## Methods

### Study design

A randomized-controlled trial (RCTs) was performed in our study. Three observation time points were set in each group. The main aims of this study were to investigate the polarization of Liver macrophages which were isolated from the rats with acute pancreatitis and to evaluate the variability and plasticity of the polarization of Liver macrophages in vitro.

### Animal model and related treatment

This animal study was approved by the Animal Center of Tongji Medical College and the Science and Research Office in Wuhan Union Hospital of Tongji Medical College, Huazhong University of Science and Technology. Sixty male Sprague Dawley rats (approximately 200–250 g in weight, 8 weeks old) were purchased from the Animal Center of Tongji Medical College, Huazhong University of Science and Technology. This study was conducted according to the Chinese National Animal Welfare law.

Fifteen rats (sham operation rats, CTRL) were administered with saline and the remaining 45 rats (SAP rats) were administered with 5% sodium taurocholate (1 ml/Kg, Sigma Co. China) which is the most popular material to induce SAP [19]. All administrations were performed via the pancreatic duct. The rats were all anesthetized with 1% phenobarbitone (0.4 ml/100 g), the pancreatic ducts of the rats were cannulated and the solution was injected slowly under low pressure with a temporary clamp of the common bile duct. The rats were sacrificed with CO_2_ euthanasia before their awakening from anesthesia of surgery. Liver macrophages were isolated from CTRL rats and treated with PBS, Treg or IL4 for 4 h. Liver macrophages were cultured from SAP rats and incubated with PBS, Treg or IL4 for 4 h, 8 h or 16 h (5 rats per treatment X time point).

### Isolation of CD4+CD25+ regulatory T cell (Treg)

Primary cultures of CD4+CD25+Treg cells were prepared from the spleens of healthy SD rats. The spleens were removed from anesthetized rats and homogenized with 10 ml of phosphate buffered saline (PBS). The cell suspension was centrifuged (1500 rpm; 10 min) and the pellet was resuspended in 4 ml PBS and 4 ml lymphocyte separation medium. After the solution was centrifuged (1500 rpm; 20 min), the interphase, which contains lymphocytes, was carefully aspirated by pipet. To isolate Treg cells, a total of 2×10^7^ lymphocytes were incubated with CD4+ FITC antibody (BD Co, Shanghai, China) for 30 minutes and sorted by immune magnetic beads (IMB). Subsequently, the separated cells were incubated with CD25-PE antibody (BD Co, Shanghai, China) for 30 minutes and sorted by IMB. On the first day after Treg isolation, the cells were activated with PMA (10 ng/ml, Sigma Co, Shanghai, China) and ionomycin (250 ng/ml, Sigma Co, Shanghai, China) [20].

### Primary culture of liver macrophages

At 24 hour post-operation, livers were perfused in situ by calcium- and magnesium-free Hanks balanced salt solution (HBSS, GIBCO Co, Shanghai, China) through the portal vein to remove any blood in the organ. Subsequently, HBSS containing 0.02% collagenase IV (GIBCO Co, Shanghai, China) was used to digest the liver until it had lost its firm texture. The soft livers were immersed in HBSS containing collagenase and were pipetted up and down to produce a single cell suspension. These suspensions were centrifuged at 70×g, 3 min and the pellet was washed with HBSS, centrifuged (70×g, 3 min) and resuspended 3 times. The last suspension was used to isolate Liver macrophages using a Percoll density gradient (50%: 25% = 1∶1, GE Co, Shanghai, China) by centrifugation (1800×g, 15 min). Then, the primary liver macrophage suspension was plated into 60 mm culture plates. After incubation for 1 hour, the supernatant was removed and attached cells were liver macrophages which were used in our study. The purity of liver macrophages was measured by flow cytometry using CD11b/c antibody (Ebioscience, San Diego, USA) [21].

### In vitro reversion of the polarized liver macrophages

In terms of treatment group, Liver macrophages were incubated with HBSS (control group), HBSS containing IL-4 (10 ng/ml, IL-4 group) or co-cultured with Treg cells using a transwell system (Kupffer cells: Treg cells approximately a 2∶1 ratio) as previously described [22]. Actually, we have tested different ratio (liver macrophages: Treg) at 1∶1, 2∶1 and 4∶1 before formal experiment and found 2∶1 is the most optimal ratio. 4 h,8 h or 16 h after incubations, macrophages were collected for further analysis.

### Measurement of polarization of liver macrophages

#### PCR

Real-time PCR was performed with the ABI Prism 7900HT (Applied Biosystem) using the SYBR Green PCR Master Mix (Applied Biosystems, Foster City, CA). The PCR primers used were as follows: GAPDH forward 5′-GTATGACTCTACCCACGGCAAGT-3′, reverse 5′- TTCCCGTTGATGACCAGCTT-3′; IL-1 beta, forward 5′- CAGGATGAGGACCCAAGCA- 3′, reverse 5′- CACAGAGGACGGGCTCTTCT- 3′;TNF-α forward 5′- CCGATTTGCCACTTCATACCA-3′ reverse 5′- TAGGGCAAGGGCTCTTGATG-3′; IL-6, forward 5′- CCCACCAGGAACGAAAGTCA- 3′, reverse 5′- AGTCCCAAGAAGGCAACTGG- 3′; iNOS, forward 5′- CAGCCCTCAGAGTACAACGAT -3′ reverse 5′- CAGCAGGCACACGCAATGAT -3′; IL-10, forward 5′- AGAAGGACCAGCTGGACAACAT -3′ reverse 5′- CAAGTAACCCTTAAAGTCCTGCAGTA -3′; TGF-β1, forward 5′- AACTACTGCTTCAGCTCCAC -3′ reverse 5′- TGTGTCCAGGCTCCAAATGTA -3′; CD163, forward 5′- CTGGGATGTCCAACTGCCAT -3′ reverse 5′- AATGCTTCCCCCATTCCTGG -3′; and Arg-1, forward 5′- CCGCAGCATTAAGGAAAGC -3′, reverse 5′- CCCGTGGTCTCTCACATTG-3′.

### Immunofluorescence

Liver macrophages were isolated for immunofluorescence staining of IL-1 and IL-10 by seeding them into 6-well plates and incubating with different treatments. After incubation, the cells were fixed with cold methanol: acetone (1∶1) for 10 min on ice. After washing the cells with PBS (pH 7.2) and blocking in solution containing 1% immunohistochemical-grade bovine serum albumin (BSA; Vector Laboratories, Burlingame, CA, U.S.A) the cells were incubated overnight with an IL-1 primary antibody (Santa Cruz biotechnology, Dallas, USA) and IL-10 primary antibody (Abcam, Cambridge, USA) at 4°C. After three washes with blocking solution, the sections were incubated for 30 min at room temperature with secondary antibodies (Antgene, Wuhan, China) and viewed by confocal laser microscopy.

### Statistics

All values were expressed as means±S.E. Independence t-test was used in the comparison of two groups. A p value <0.05 was determined to indicate statistical significance. All analysis was calculated by SPSS for windows 17.0.

## Results

### Isolation of regulatory T cells and liver macrophages

**Figure 1:**
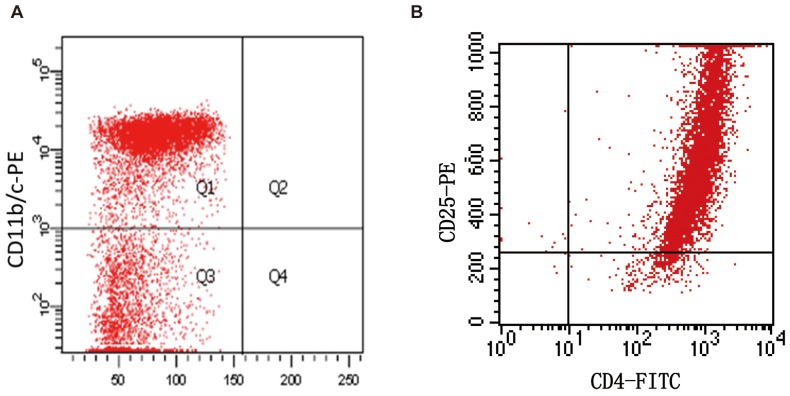
Identification of regulatory T cells and liver macrophages using flow cytometry. **A:** liver macrophages were identified as CD11b/c positive cells after primary culture for 24 hours. **B:** The purity of Treg cells was examined after isolation using magnetic beads.

Treg cells and liver macrophages were isolated from SD rats and cultured as described in the methods. After primary culturing, the Treg cells and macrophages were examined for the purity. The purity of liver macrophages was approximately 75% and that of Treg cells was approximately 90% (Fig. 1).

### Induction of acute pancreatitis

**Figure 2:**
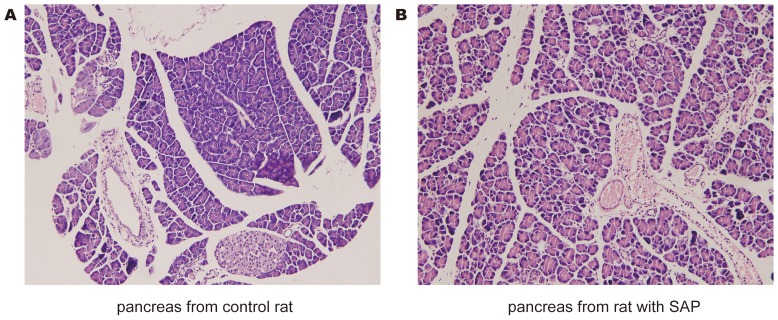
Confirming induction of the severe acute pancreatitis model. Assessment of pancreatic injury by HE staining. Pancreatitis was induced by pancreatic-ductal perfusion with 5% Sodium taurocholate. All rats were sacrificed at 24 h after disease model initiation. A: A typical photomicrographs of HE stained tissue obtained from control rat. B: A photomicrograph of pancreatic injury which includes pancreatic edema, neutrophil infiltration, necrosis and hemorrhage.

Experimental acute pancreatitis was induced by infusing 5% Sodium taurocholate into the pancreatic duct. As shown in Fig. 2, the morphology of the pancreas in control rats appeared almost normal. However, in SAP rats, the morphology of pancreas demonstrated necrosis, bleeding and leukocyte infiltration.

### Demonstration of M1 polarization of liver macrophages during acute pancreatitis

Liver macrophages were isolated from the rats in the control and SAP groups. The expression of major M1 markers (IL-1, TNF-α, iNOS and IL-6) and M2 markers (IL-10, TGF-β1, CD163 and Arg-1) were evaluated using real time PCR. IL-1 and IL-10 were observed with immunofluorescence methods.

**Figure 3:**
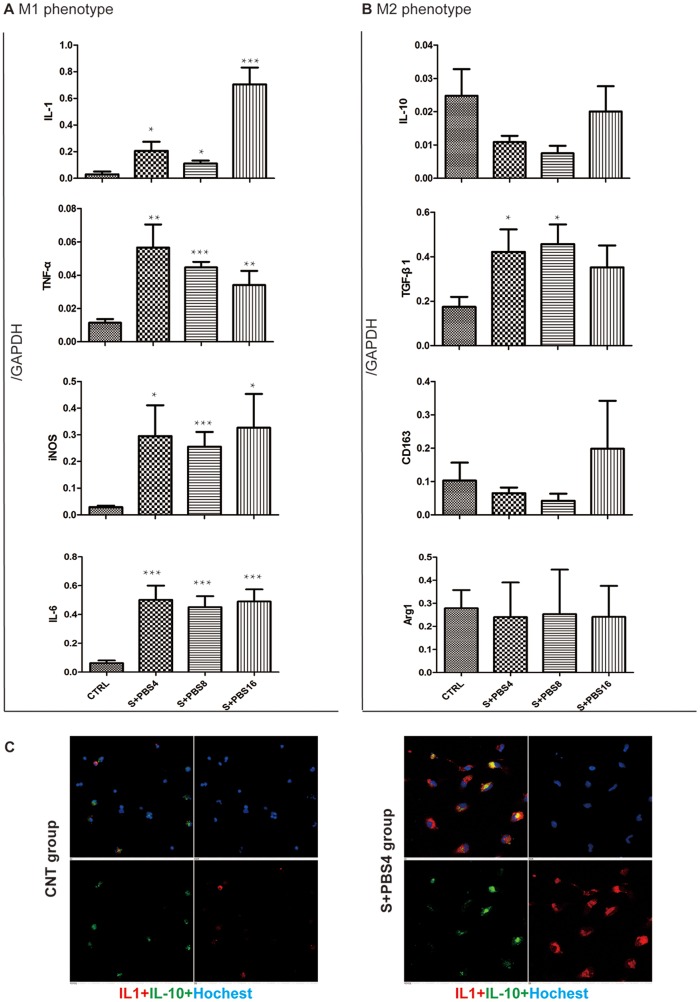
Demonstration of M1 polarization of Liver macrophages isolated from the rats with acute pancreatitis. Liver macrophages were obtained from CTRLthe rats post operation for 24 hours (all other groups). In vitro, macrophages from SAP rats treated with PBS for 4, 8 and 16 hours respectively (S+PBS4, S+PBS8, S+PBS16 group). and macrophages from CTRL rats undergone sham operation were treated with PBS for 4 h. The polarization of M1/M2 was assessed using real time PCR and immunofluorescence. A: mRNA level of M1 markers in the macrophages. B: mRNA level of M2 markers in the macrophages. C: Immuno-fluorescence examination of macrophage polarization. The significance of differing mRNA levels was analyzed while comparing with the CTRL group. Error bars indicate the mean ±S.E. *p<0.05, **p<0.01, ***p<0.001.

As shown in Fig. 3, the results of real-time PCR indicated that the mRNA levels of M1 markers were all significantly higher in the SAP groups than in the control group (p value all <0.05). In contrast, mRNA levels of M2 markers except TGF-β1 in SAP groups were similar to those in the control group (p value all >0.05). TGF-β1 is upregulated during the course of SAP. Immunofluorescence analysis also showed that macrophages from rats with SAP expressed more IL-1 and less IL-10 than macrophages from the control rats. Taken together, these results suggest a shift toward M1 polarization of liver macrophages during SAP.

### Reverse the polarization of liver macrophages in vitro

Next we investigated whether the polarization of liver macrophages from SAP rats could be reversed to M2 state in vitro. We compared the level of M1/M2 marker expression in primary culture cells, at the same 8 h time point, after different treatments (PBS, IL-4 and Treg cells).

**Figure 4:**
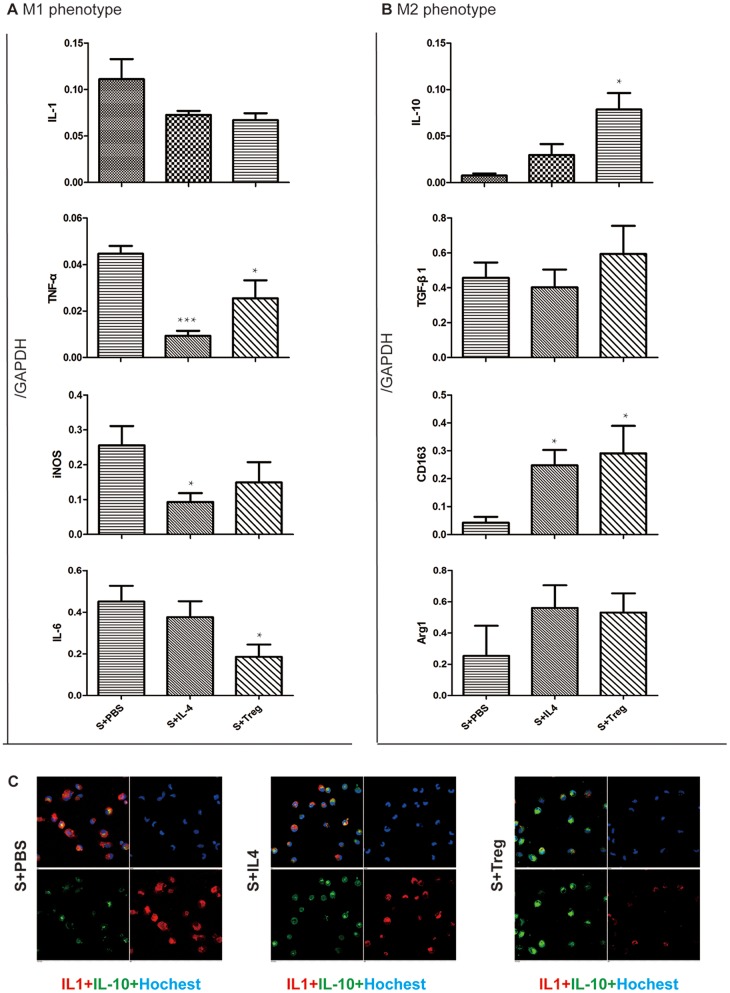
Reverse the polarization of liver macrophages by IL-4 and Treg in vitro. Liver macrophages were obtained from at 24 h after SAP model initiation. In vitro, macrophages were respectively treated with PBS, IL-4 (10 ng/ml) or Treg (macrophages: Treg ≈2∶1) for 8 hour (S+PBS, S+IL-4, S+Treg group). The M1/M2 polarization was assessed using real-time PCR and immunofluorescence. A: mRNA level of M1 markers in the macrophages. B: mRNA level of M2 markers in the macrophages. C: Immunofluorescence examination of the polarization of macrophages. The significance of differing mRNA levels was analyzed while comparing with the CTRL group. Error bars indicate the mean ±S.E. *p<0.05, **p<0.01, ***p<0.001.

As shown in Fig. 4, the results of real-time PCR indicated M1 marker down-regulated after the treatment of IL-4 or Treg and some difference of them were significant (p value <0.05). In contrast, M2 polarization markers, especially CD163 and IL-10 but not TGF-β1, were upregulated by IL-4 or Treg treatment. These results suggested that both IL-4 and Treg can reverse the polarization of liver macrophages from M1 to M2 phenotypes.

### Comparing the effect of IL-4 and Treg cell treatment on reversing macrophage polarization

To determine the better approach for altering the polarization of macrophages in vitro, we compared the expression of M1 and M2 markers after the treatment of IL-4 or Treg cells.

**Figure 5:**
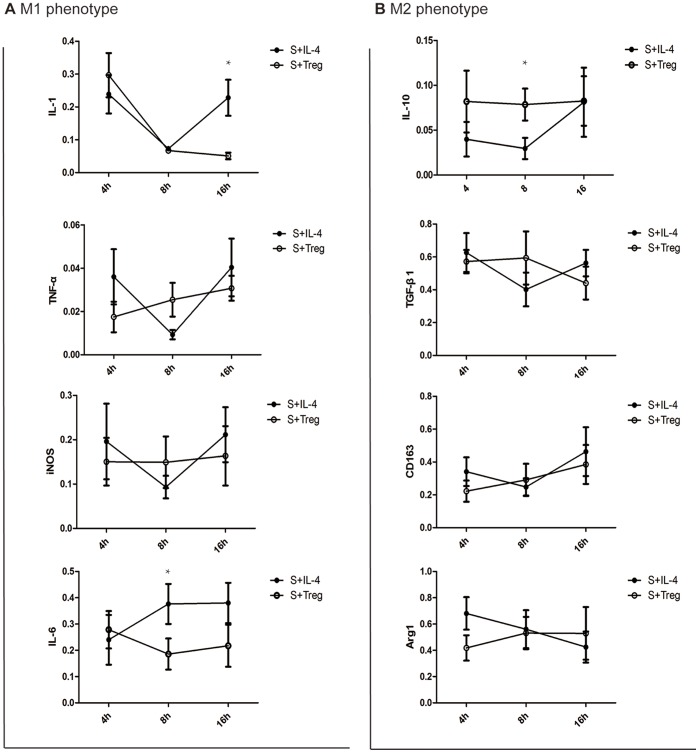
Treg cells reversed the polarization of Liver macrophages longer than IL-4 treatment. In vitro, macrophages were treated with IL-4 (10 ng/ml) or Treg (macrophages: Treg ≈2∶1) for 4, 8 and 16 hour. The polarization of M1/M2 was assessed using real time PCR and immunofluorescence. A: mRNA level of M1 markers in the macrophages. B: mRNA level of M2 markers in the macrophages. The significance of differing mRNA levels was analyzed while comparing the two groups in the same time point using the indifferent T-test. Error bars indicate the mean ±S.E. *p<0.05.

Gene expression in primary culture macrophages from the rats with acute pancreatitis was determined by real-time PCR. As shown in Fig. 5, the effect of treatment with IL-4 and Treg cells were similar (p value >0.05) except that some groups that the level of IL-1 (16 h) and IL-6 (8 h) were much lower and IL-10 (8 h) were significant higher after Treg treatment comparing to that in IL-4 treatment group.

## Discussion

Severe acute pancreatitis is a type of acute inflammation with high patient mortality. Overexpression of cytokines such as IL-23, TNF-α, MCP-1 is widely accepted as being indispensable in the occurrence of MODS [4]; [5]; [23]. Furthermore, increasing numbers of reports have demonstrated that these cytokines, which contribute to MODS in acute pancreatitis, largely originated from macrophages, especially liver macrophages [8]; [9]; [24].

There are two kinds of functional or polarized states of macrophages. M1 polarization is the classic functional subtype of macrophages, and these macrophages can produce activated proinflammatory mediators such as IL-1 or TNF-α which contribute to severe inflammation. In contrast, M2 polarized macrophages produce some regulatory cytokines such as IL-10 which can down-regulate traditional systemic immune reactions [10]; [11]; [12].

In our work, we found that liver macrophages were M1 polarized after the induction of acute pancreatitis which was confirmed by the up-regulated expression of M1 markers and no alteration of expression of M2 markers. This M1 polarization could be observed from 4 hours after the onset of acute pancreatitis and was present at 16 hours. In addition, a high level of TGF-β_1_ may be related to the apoptosis of pancreatic acinar cells during SAP [24]. These observations agree with the conventional theory that macrophages produce proinflammatory cytokines and amplify the level of systemic inflammation during acute pancreatitis [3]; [8].

The liver macrophages can be activated after they engulf necrotic tissue and endotoxin which often exist in the blood of patients with acute pancreatitis. This results in the activation of M1 polarized macrophages which leads to an increase in the intensity of inflammation. However, several reports showed that M1 polarization of macrophages could be reversed to M2 polarization [25]; [26]. We hypothesized that liver macrophages could not aggravate inflammation but alleviate the immune response if we reversed the polarization of liver macrophages in acute pancreatitis.

In this study, we first attempted to reverse the polarization of liver macrophages in vitro. We isolated liver macrophages from the rats with acute pancreatitis and the isolated cells were treated by IL-4 or Treg cells. Our data showed that both treatments can reverse M1 polarization toward an M2 state. After treatment, liver macrophages down regulated the expression of M1 markers such as IL-1, IL-6, TNF-α and iNOS which are also pro-inflammatory mediators. Concurrently, macrophages produced more M2 markers such as IL-10 which is an anti-inflammatory agent. In this sense, our study indicates that M1 polarization of macrophages can be reversed after treatment of IL-4 or Treg. Furthermore, this kind of reversion may decrease systemic inflammation and promotes tissue repair during acute pancreatitis.

There is some evidence from other studies that IL-4 can reverse the polarization of macrophages [25]; [27]; [28]. Our results are consistent with these early supports. In addition, in our study, Treg cells demonstrated their capability to alter the polarization of macrophages toward the M2 phenotype. We propose that this occurs because activated Treg cells can produce IL-4. We hypothesized that the effect of treatment with Treg cells may persist longer than that of IL-4 because of the short IL-4 half-life. Our results show that although the liver macrophages after Treg cell treatment secreted more IL-10 and less IL-1 and IL-6 at similar time points than the macrophages treated by IL-4, the effect of these two treatments was very similar. In consideration of the existence of CD4+CD25+/Treg- cells, the Treg we used in this study is not as much as it showed in the Flowcytometry. Therefore, we speculate that the ability of reversing macrophage polarization by pure Treg cell treatment would be much better than that of IL-4 because IL-4 might be eliminated more quickly in vivo than in vitro.

In summary, we demonstrate here that liver macrophages present a M1 polarization after the occurrence of acute pancreatitis. IL-4 and Treg cells can shift the polarization of macrophages from M1 to M2 in vitro. Furthermore, we will next verify the effect of IL-4 and Treg cells on the reversal of macrophage polarization in vivo, and we hypothesize that Treg cells are more advantageous because they can make persistent effects. This advantage of Treg is therapeutically important because it can overcome the difficulty of IL-4 application in clinic because of short half-life.
